# Immune checkpoint TIM-3 in tumor immunotherapy

**DOI:** 10.3724/abbs.2025235

**Published:** 2025-12-24

**Authors:** Shuaiya Ma, Mengyao Zhu, Chunhong Ma, Chunyang Li

**Affiliations:** 1 The Affiliated Cancer Hospital of Zhengzhou University & Henan Cancer Hospital Zhengzhou 450008 China; 2 Key Laboratory for Experimental Teratology of Ministry of Education and Department of Immunology School of Basic Medical Sciences Cheeloo College of Medicine Shandong University Jinan 250012 China; 3 Key Laboratory for Experimental Teratology of the Ministry of Education and Department of Histology and Embryology School of Basic Medical Sciences Cheeloo College of Medicine Shandong University Jinan 250012 China

**Keywords:** TIM-3, immune checkpoint, immunotherapy

## Abstract

Over the past decade, immunotherapy has emerged as a pivotal therapeutic strategy in cancer treatment. Immune checkpoint inhibitors (ICIs), such as CTLA-4 and PD-1 monoclonal antibodies, have demonstrated remarkable clinical efficacy in different types of cancer. However, the overall success rate of immune checkpoint therapies remains low. Investigating alternative immune checkpoint molecules is imperative. T-cell immunoglobulin and mucin-containing molecule-3 (TIM-3), which is expressed in T cells, natural killer (NK) cells, macrophages, and dendritic cells, has gained recognition as a promising candidate for tumor immunotherapy. Targeting TIM-3 represents a promising approach for cancer immunotherapy, particularly through the rational design of novel combination therapies with other ICIs. In this review, we present a comprehensive summary of the research advancements concerning the role of TIM-3 in regulating immune responses in different cell types and explore theoretical frameworks for targeting TIM-3 to achieve more effective immunotherapeutic breakthroughs.

## Introduction

Immunotherapy involving immune checkpoint inhibitors (ICIs) has become a critical approach for combating cancer. The United States Food and Drug Administration (FDA) has approved anti-CTLA-4 monoclonal antibodies (mAbs) and anti-PD-1 mAbs for the treatment of melanoma and non-small cell lung carcinoma (NSCLC) [ 1– 3] . Although ICI therapies have improved survival compared with traditional cancer treatments for various cancer types, only 10%-30% of patients benefit from these therapies in most cancer types [4]. Therefore, it is imperative to identify new immune checkpoint molecules and elucidate their underlying mechanisms to further advance cancer immunotherapy.

T-cell immunoglobulin and mucin-containing molecule-3 (TIM-3) was initially identified in 2002 and is expressed on CD4 ^+^ T helper 1 (Th1) and CD8 ^+^ cytotoxic T lymphocytes (CTLs) [5]. Subsequent studies revealed that TIM-3 is also expressed on the surface of activated natural killer (NK) cells [6], Th17 cells [7], gamma delta (γδ) T cells [ 8– 11] , regulatory T cells (Tregs) [12], macrophages [5], dendritic cells (DCs) [13], and mast cells [14]. TIM-3 belongs to the TIM gene family, which in humans comprises three members: TIM-1, TIM-3, and TIM-4. These proteins are encoded by the genes *HAVCR1*, *HAVCR2*, and *TIMD4*, respectively [15]. TIM-3 consists of an immunoglobulin variable (IgV) domain, a mucin stalk domain, a single transmembrane domain, and a cytoplasmic tail domain [16]. It has at least four ligands: galectin-9 (Gal-9), carcinoembryonic antigen cell adhesion molecule 1 (CEACAM1), high-mobility group protein B1 (HMGB1), and phosphatidylserine (PtdSer), all of which interact with the TIM-3 IgV domain. The interaction between TIM-3 and its ligands mediates the suppression of anti-tumor immune responses [17]. Gal-9, the first ligand identified, induces apoptosis in Th1 cells and negatively regulates Th1 cell immunity [ 18, 19] . Furthermore, the TIM-3/CEACAM1 interaction induces T cell exhaustion [20]. Numerous preclinical studies across various tumor types have demonstrated that TIM-3 blockade can inhibit tumor progression, particularly when combined with PD-1/PD-L1 blockade [ 21, 22] . Therefore, targeting TIM-3, either in combination with other ICIs or through integrating TIM-3 inhibition with novel immunotherapeutic strategies that activate cancer-specific T-cell stimulatory molecules, shows great promise for advancing therapeutic approaches with enduring clinical efficacy.

In this review, we focus on the biological characteristics and functions of TIM-3 in cancer immunology, elucidate the underlying mechanisms, and review key findings from clinical studies.

## Research History and Key Milestones of TIM-3

McIntire and colleagues identified the mouse TIM gene family through genomic analysis, mapping it to chromosome 11, which encodes transmembrane glycoproteins with IgV and mucin domains [23]. Subsequently, Monney *et al*. [5] were among the first to identify TIM-3, which contains an immunoglobulin and a mucin-like domain and is expressed on differentiated Th1 cells. Th1-specific cell surface protein TIM-3 modulates the severity of autoimmune diseases by regulating macrophage activation and/or function. Gal-9 was first identified as the ligand of TIM-3, and its binding leads to Th1 cell apoptosis while inhibiting immune responses [18]. DeKruyff *et al*. [24] demonstrated that TIM-3 recognizes PtdSer and mediates the phagocytosis of apoptotic cells. These findings establish a novel paradigm, suggesting that TIM-3 functions as a PtdSer receptor and consolidates the functional understanding of TIM-3.

As the structure, functions, and ligands of TIM-3 were discovered, a growing body of evidence has revealed the roles of TIM-3 in various diseases, highlighting it as a critical therapeutic target. Sakuishi *et al*. [21] reported that TIM-3 is highly expressed on PD-1 ^+^ tumor-infiltrating lymphocytes (TILs). These TIM-3 ^+^PD-1 ^+^ TILs exhibit the most severe exhausted phenotype, which is characterized by impaired proliferation and production of IL-2, TNF-α, and IFN-γ. Targeting the TIM-3 and PD-1 pathways simultaneously has been reported to reverse T-cell exhaustion and restore anti-tumor immunity. In the same year, Ju *et al*. [25] reported elevated expression levels of TIM-3 on NK cells in patients with chronic hepatitis B (CHB). Moreover, blocking the TIM-3 pathway significantly augmented the cytotoxic activity of NK cells in CHB patients. Increasing studies have expanded the known ligand repertoire of TIM-3, demonstrating that its interaction with CEACAM1 and HMGB1 promotes tumor evasion by blocking antigen presentation or suppressing innate immunity [ 13, 20] .

**Figure 1:**
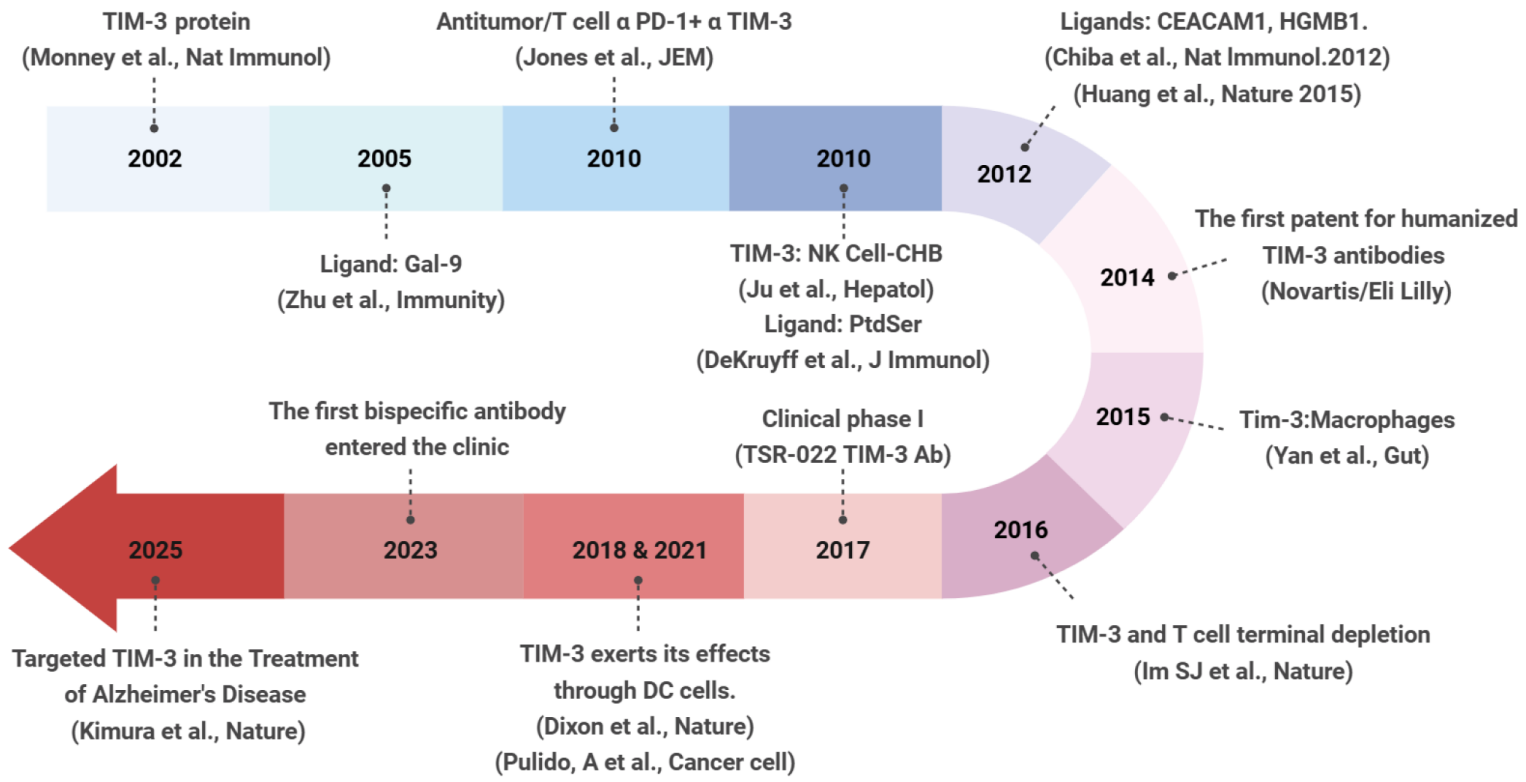
Figure 1 Timeline of TIM-3 research development This circular timeline chronicles pivotal advances in TIM-3-related research—spanning molecular biology, ligand characterization, and translational therapeutic development—over the period from 2002 to 2025.

Based on a comprehensive understanding of the ligands and functions of TIM-3, Novartis developed MBG453, the first humanized monoclonal antibody specifically targeting TIM-3. In addition to T and NK cells, Yan *et al*. [26] demonstrated that TIM-3 is highly expressed on both peripheral blood monocytes and tumor-associated macrophages (TAMs) in patients with hepatocellular carcinoma (HCC). Targeting TIM-3 significantly inhibited the alternative activation of macrophages and suppressed the growth of HCC cells, suggesting that interference with TIM-3 may hold significant potential for HCC treatment. Im *et al*. [27] reported that terminally exhausted CD8 ^+^ T cells presented high expression of TIM-3 in chronic infections and cancer. TSR-022 was the first TIM-3 humanized monoclonal antibody evaluated in phase I clinical trials, with no dose-limiting toxicity observed. Several studies have increasingly focused on the expression of TIM-3 on DCs. Mingo *et al*. [28] demonstrated that TIM-3 is highly expressed on CD103 ^+^ DCs and that an anti-TIM-3 antibody regulates the function of CD103 ^+^ DCs and their response to paclitaxel chemotherapy in models of triple-negative and luminal B disease. Dixon *et al*. [29] further elucidated the critical role of TIM-3 in regulating DC function and reported that TIM-3 blockade enhances antitumor immunity by regulating inflammasome activation. AZD7789 was the first reported dual TIM-3/PD-1 antibody applied in phase I/II clinical trials [30]. Currently, research on TIM-3 has expanded to a wider range of diseases. Kimura *et al*. [31] revealed that TIM-3 is involved in maintaining microglial homeostasis through TGFβ signaling, underscoring the therapeutic potential of targeting microglial TIM-3 in Alzheimer’s disease (AD) ( Figure 1).

### The structure of TIM-3

**Figure 2:**
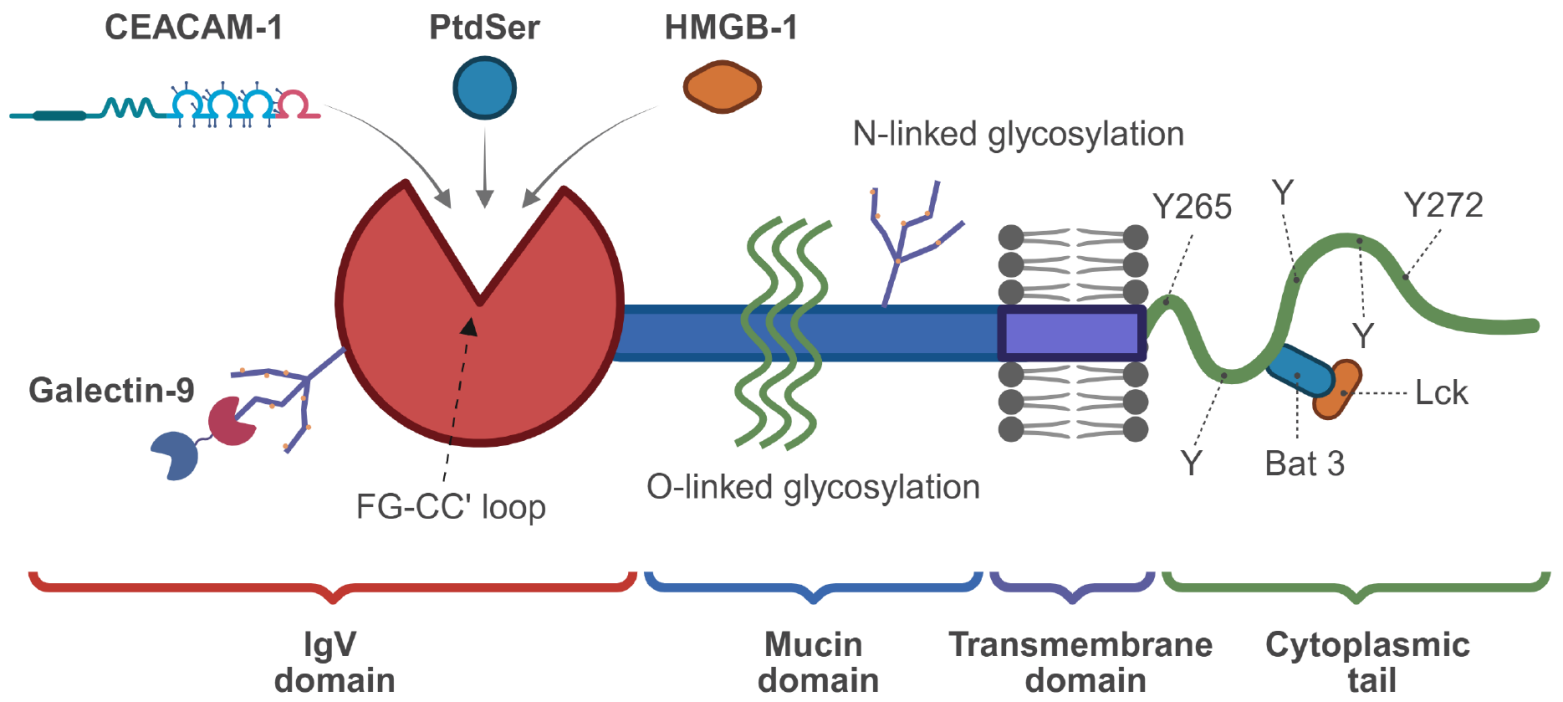
Figure 2 Diagram illustrating the structure of TIM-3 and its ligand interaction pattern This schematic outlines TIM-3’s domain organization, key ligands, and intracellular interactors: (1) Extracellular domains: the N-terminal IgV domain harbors the FG-CC′ loop (core ligand-binding cleft), mediating binding to four ligands (Galectin-9, CEACAM-1, PtdSer, HMGB-1), while the adjacent mucin domain has O-linked glycosylation and the N-linked glycosylation also occurs extracellularly; (2) Transmembrane domain: membrane-embedded, anchoring the protein; (3) Cytoplasmic tail: contains tyrosine residues (e. g., Y265, Y272) and interacts with Bat3/Lck, regulating TIM-3’s downstream signaling.

Structurally, TIM-3 is a type I transmembrane protein with four domains: an IgV domain with N-linked glycosylation sites and a conserved FG-CC′ cleft critical for its function, a mucin stalk domain with both N-linked and O-linked glycosylation sites contributing to its diversity, a transmembrane domain, and a cytoplasmic tail ( Figure 2)[ 16, 32, 33] .

**Figure 3:**
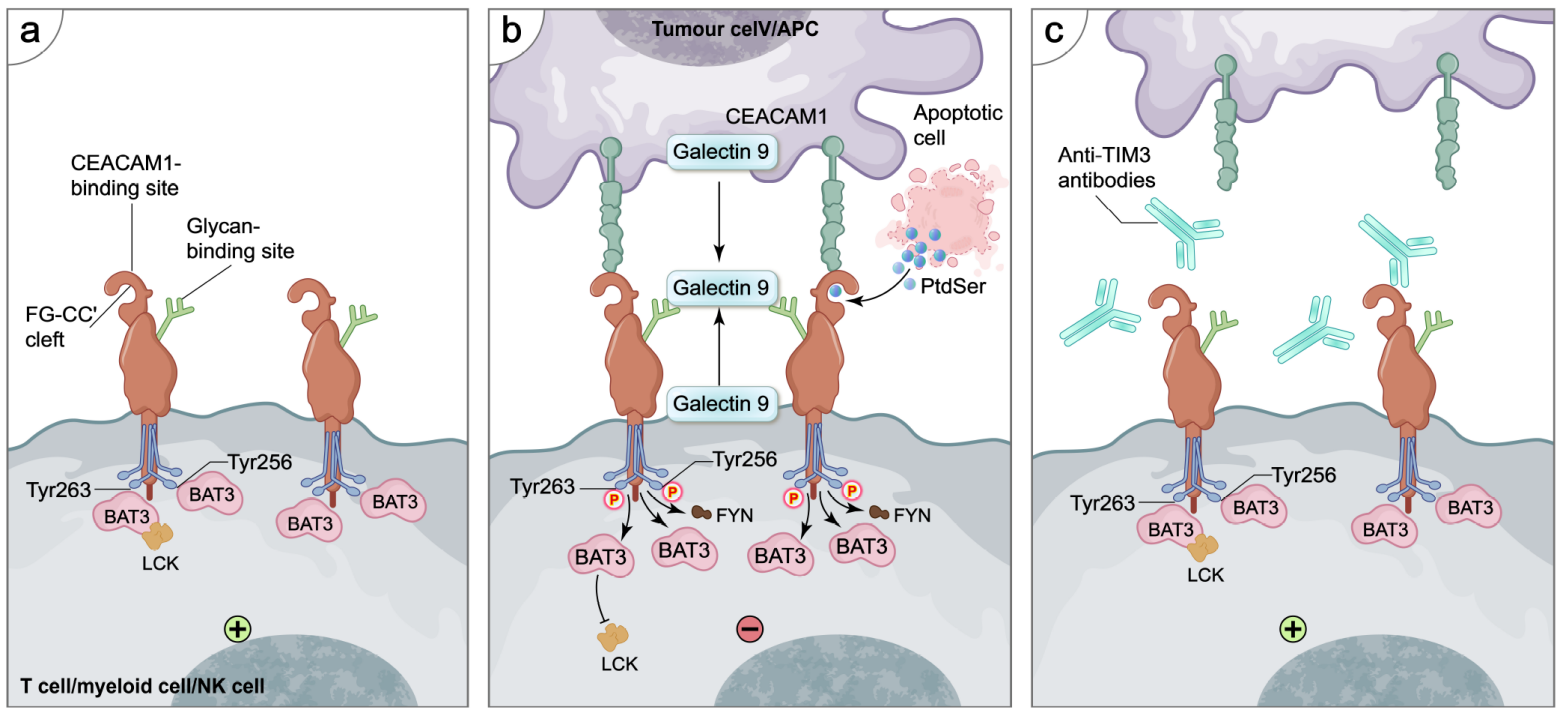
Figure 3 Interaction models of TIM-3 and ligands This diagram depicts TIM-3’s functional states and targeted intervention in immune cells (T cells, myeloid cells, NK cells). a (basal state): membrane-localized TIM-3 (via FG-CC′ cleft/intracellular domain) interacts with BAT3/LCK, supporting basal immune signaling (marked “+”). b (immunosuppressive activation): tumor cells/APCs present TIM-3 ligands (Galectin 9, CEACAM1, apoptotic PtdSer). Ligand binding induces Tyr256/Tyr263 phosphorylation, dissociating BAT3 and recruiting FYN to transmit inhibitory signals (marked “−”), suppressing immune activity. c (therapeutic targeting): anti-TIM3 antibodies block ligand binding, restoring TIM-3’s BAT3/LCK interaction and reactivating immune signaling (marked “+”).

The TIM-3 gene is located at locus 5p33.3 in humans and chromosome 11B1.1 in mice [34]. The human TIM-3 protein is composed of 301 amino acids, whereas its murine counterpart consists of 281 amino acids, exhibiting 63% sequence homology [35]. The IgV domain of TIM-3 presents four noncanonical cysteines, which form two unique disulfide bridges essential for ligand binding [36]. In conventional IgV models, the FG and CC′ loops are situated on opposing sides, approximately 25 Å apart. In contrast, in TIM-3, these unique disulfide bonds reorient the CC′ loop in closer proximity to the FG loop, thereby creating a distinctive “cleft” in TIM-3 and a small associated channel with dimensions of approximately 7.5 × 9.5 × 11.0 Å ^3^. The cleft formed by the CC′ and FG loops is stabilized not only by the two noncanonical disulfide bonds but also by a series of conserved ionic and hydrogen bonding interactions [ 36, 37] . The FG-CC′ cleft of TIM-3 possesses a metal ion-dependent ligand binding site, which is chelated by Ca ^2+^ and engages in interactions with PtdSer and CEACAM1. The Ca ^2+^-binding site of human TIM-3 (hTIM-3) is located within the F-G loop and comprises the residues Ile114, Gly116, Asn119, and Asp120, which collaborate to coordinate a single Ca ^2+^ cation. Specifically, the main-chain oxygen atoms of Ile114 and Gly116, along with the side-chain oxygen atoms of Asn119 and Asp120, form equidistant bonds (2.3 Å) with the bound Ca ^2+^ ion. Consequently, two additional sites remain open for ligand-binding coordination. Notably, the residues Asn119 and Asp120, which interact with Ca ^2+^ via their side chains, are conserved throughout all human TIM family members. PtdSer interacts with the residues Asn119 and Asp120 in the F-G loop of hTIM-3 through calcium ion-mediated binding. CEACAM1 interacts with the residues Glu62 and Arg69 in the C-C loop of hTIM-3 [38]. Gal-9 has been characterized as a TIM-3 ligand with the specific capability of recognizing carbohydrate motif(s) present on the TIM-3 IgV domain, inducing calcium influx and cell death [18]. The cytoplasmic tail of TIM-3 lacks the inhibitory ITIM motif but contains five conserved tyrosine residues that are crucial for signal transduction. Specifically, Tyr265 (Tyr256 in mice) and Tyr272 (Tyr263 in mice) can interact with HLA-B-associated transcript 3 (BAT3) [39] and the protein tyrosine kinase FYN [40], respectively. In the absence of ligand binding, BAT3 interacts with the Y256/Y263 residues in the cytoplasmic tail of TIM-3. This interaction recruits the Src kinase Lck and promotes its activity. Consequently, increased Lck activity facilitates the recruitment of ZAP70, thereby promoting TCR activation. Ligand binding triggers the phosphorylation of Tyr265/272 by ITK, leading to the dissociation of BAT3 and the recruitment of FYN to transmit inhibitory signals. The interaction between BAT3 and TIM-3 ultimately induces T-cell exhaustion [ 20, 34] . Therefore, the interaction between TIM-3 and its cognate molecules plays a pivotal role in finely tuning the balance between T-cell activation and exhaustion. Anti-TIM-3 antibodies can block the interaction between ligands and TIM-3, inhibit tyrosine phosphorylation, and restore LCK recruitment, thereby exerting anti-tumor effects ( Figure 3).

Additionally, there is a soluble form of TIM-3, known as sTIM-3 (soluble TIM-3). In mice, this soluble form may be generated through alternative splicing at the mRNA level. Indeed, murine studies aimed at characterizing sTIM-3 demonstrated that alternatively spliced constructs can extrinsically suppress T-cell responses [ 41, 42] . Research has indicated that the production of human soluble TIM-3 is mediated by the shedding of cell-surface molecules at the protein level, which is induced by matrix metalloproteinases (MMPs), specifically ADAM10 and ADAM17 [43]. Previous studies have demonstrated that sTIM-3 undergoes ectodomain shedding from CD8 ^+^ T cells mediated by the sheddase enzyme ADAM10. Notably, they reported that plasma levels of sTIM-3 are significantly elevated during untreated HIV infection and are positively correlated with the progression of HIV disease [ 43, 44] . Moreover, sTIM-3 has been reported to hinder T-cell-mediated antitumor responses. Hui *et al*. [42] reported that overexpression of sTIM-3 in C57BL/6 mice inoculated with B16F1 melanoma cells promoted tumor growth by inhibiting T-cell activity and reducing infiltration.

sTim-3 has significant clinical value in predicting the treatment efficacy and prognosis of various cancers. Conde-Rodríguez *et al*. [45] demonstrated a correlation between sTIM-3 levels and the progression and clinical stage of cervical cancer, with elevated sTIM-3 levels being associated with more advanced clinical stages. Additionally, plasma sTIM-3 is a promising blood-based biomarker associated with ICI efficacy in mccRCC, with differential effects on nivolumab versus N + I (nivolumab + ipilimumab), and is independent of common clinical or inflammatory markers [46]. Our laboratory previously reported an increase in serum sTIM-3 in patients who did not respond to anti-PD-1 therapy for non-small cell lung cancer (NSCLC) and in those with anti-PD-1-resistant cholangiocarcinoma [47]. Mechanistically, sTIM-3 functions as a ligand of CEACAM1 on T cells, which not only induces T-cell exhaustion but also dampens the responsiveness of CD8 ^+^ T cells to PD-1 blockade therapy. Consequently, sTIM-3 has emerged as a promising predictive biomarker and a potential therapeutic target within the realm of tumor immunology [47]. Therefore, by monitoring the dynamic changes of sTIM-3, treatment plans can be adjusted in a timely manner to improve patient prognosis.

### Ligand and signaling of TIM-3

To date, four ligands have been shown to interact with the IgV domain of TIM-3. These ligands include Gal-9, HMGB1, CEACAM1, and PtdSer. The interaction mechanisms between TIM-3 and these ligands, along with their biological implications, are described in the following subsections.

#### Galectin 9

Gal-9, the first identified ligand of TIM-3, has a molecular weight of 35 kDa and specifically binds to carbohydrate motifs on the IgV domain of TIM-3. This interaction facilitates the oligomerization of TIM-3 on the cell surface, resulting in the phosphorylation of Tyr256 and Tyr263 within the intracellular domain of TIM-3. The TIM-3/Gal-9 interaction pathway induces intracellular calcium flux in Th1 cells, leading to their apoptosis [18]. A substantial body of evidence underscores that the inhibitory or stimulatory effects elicited by the TIM-3/Gal-9 interaction are dependent upon the specific cell type expressing TIM-3. The TIM-3/Gal-9 interaction inhibits immune responses mediated by Th1, Th17, and Tc1 cells. This inhibition is achieved through the suppression of Lck-mediated phosphorylation of the TCR and the production of IFN-γ, TNF-α, and IL-2 [ 48, 49] . Ju *et al*. [50] reported that TIM3 ^+^ Treg cells possess a more potent suppressive function than TIM3 ^-^Treg cells do and that targeting the TIM-3/Gal-9 pathway reduces their suppressive capacity. Michelle *et al*. [51] reported that the TIM-3/Gal-9 interaction enhances IFN-γ production in NK92 cells, indicating that TIM-3 serves as a coreceptor on NK cells to increase IFN-γ production. Our previous study indicated that the TIM-3/Gal-9 interaction inhibits the cytotoxicity of NK cells [25]. Additionally, TIM-3 may upregulate Gal-9 expression through a mechanism that has not been elucidated. Notably, compared with wild-type controls, transgenic mice that overexpress TIM-3 on T cells exhibit a significant increase in the frequency of Gal-9-expressing CD11b ^+^ myeloid-derived suppressor cells (MDSCs) [19]. Notably, TIM-3/Gal-9 interactions are not always immunosuppressive. In the context of tuberculosis, T cells in the lung express TIM-3, interact with Gal-9 ^+^ macrophages, and effectively restrict bacterial growth [52]. Furthermore, TIM-3/Gal-9 interactions in dendritic cells exhibit functional synergy with Toll-like receptor (TLR)-mediated innate immune activation, resulting in enhanced translocation of nuclear factor kappa-light-chain-enhancer of activated B cells (NF-κB) and subsequent amplification of inflammatory cytokine secretion [53]. Further research is warranted to elucidate the precise molecular mechanisms governing TIM-3/Gal-9-mediated regulation of both innate and adaptive immune responses.

#### CEACAM1

CEACAM1, the most recently identified ligand of TIM-3, has a molecular weight of 60 kDa and binds specifically to the conserved CC′ and FG loops of TIM-3. CEACAM1 and TIM-3 form a specific heterodimer in *trans* that is facilitated by interactions between their structurally analogous membrane-distal IgV-like, N-terminal domains. The interaction between CEACAM1 and TIM-3 triggers the release of BAT3 from the cytoplasmic tail of TIM-3, thereby activating TIM-3-mediated inhibition of TCR signaling. Additionally, CEACAM1 has been reported to enhance the glycosylation and protein stability of TIM-3. The *cis* interaction between CEACAM1 and TIM-3 contributes to the stabilization of mature TIM-3 on the cell surface [ 20, 39] . Furthermore, CEACAM1 and TIM-3 can bind in *cis* and *trans* configurations, both of which induce CD8 ^+^ T-cell exhaustion in myelodysplastic syndrome [54], suggesting that both *cis* and *trans* interactions mediate TIM-3 function. However, Leung *et al*. reported [55] that CEACAM1-deficient mice exhibit an increased tumor burden in colorectal cancer, suggesting that CEACAM1 may play a role in suppressing tumorigenesis progression. Therefore, TIM-3/CEACAM1 interactions in the tumor microenvironment are mechanistically complex and require detailed molecular characterization to clarify their regulatory networks.

#### PtdSer

PtdSer, a phospholipid that serves as a surface marker for apoptotic cells, binds to the IgV domains of TIM-1, TIM-3, and TIM-4 [ 24, 56] . Specifically, PtdSer binds to the FG-CC′ cleft of the TIM-3 IgV domain, with calcium binding coordinated by the amino acids Asn119 and Asp120 [36]. Research by Nakayama *et al*. demonstrated that the TIM-3/PtdSer interaction facilitates the uptake of apoptotic cells and mediates antigen cross-presentation [57]. Furthermore, Pagliano *et al*. [58] revealed that TIM-3 ^+^ antigen-presenting cells (APCs) engage with PtdSer on CD8 ^+^ TILs and tumor antigen-specific CD8 ^+^ T cells, which limits anti-tumor immunity through T-cell trogocytosis. We showed that the interaction of TIM-3 with PtdSer in TIM-3 ^high^ NK cells suppresses their cytotoxicity via AKT/mTORC1 signaling, thereby impairing tumor immunosurveillance [59]. Both murine and human anti-TIM-3 antibodies, which have shown functional efficacy *in vivo* and *in vitro*, disrupt TIM-3 binding to PtdSer and CEACAM1 but not to Gal-9 [60]. Therefore, the PtdSer binding pocket of TIM-3 represents a promising target for the development of TIM-3 inhibitors.

#### HMGB1

HMGB1, a DNA-binding protein, is a member of the high-mobility group family and is encoded by the *HMGB1* gene (13q12). HMGB1 functions as a chromatin-associated protein that maintains genome stability and promotes transcription. Upon cellular stress, HMGB1 translocates to the cytosol, where it mediates inflammatory responses, modulates DC differentiation, and activates NK cells [61]. Chiba *et al*. [13] reported that TIM-3 acts as a receptor for HMGB1 in DCs in tumor microenvironments. The interaction between DC-derived TIM-3 and the alarmin HMGB1 suppresses innate immune responses by recognizing nucleic acids through TLRs and cytosolic sensors. Virus-specific TIM-3 ^+^CD8 ^+^ T cells have also been shown to suppress antiviral immunity via HMGB1 recognition during both acute and chronic viral infections of the liver [62]. Although the precise binding site between HMGB1 and TIM-3 remains uncharacterized, their interaction is independent of other TIM-3 ligands.

TIM-3 plays an important immunoregulatory role via interaction with distinct TIM-3 ligands (TIM-3Ls). Multiple significant biological outcomes are elicited by the context-dependent ligand recognition mechanisms of TIM-3 ( Table 1). However, the sociological cellular dynamics mediated by the TIM-3/TIM-3L axis and its specific role in tumorigenesis remain poorly understood. Wang *et al*. [63] reported that CD4 ^+^ T cells in the vicinity of TIM-3L ^+^ cells within the tumor microenvironment exhibit markedly increased expression of TIM-3. Notably, the enhanced function of tumor-associated CD8 ^+^ T cells and NK cells induced by TIM-3-blocking antibodies is critically dependent on the presence of CD4 ^+^ T cells. CD4 ^+^ T cells play crucial roles in the sociological network of cells modulated by the TIM-3/TIM-3L interaction. This discovery not only provides a novel perspective on TIM-3-targeted cancer immunotherapy but also substantially enhances our understanding of the complex interactions between TIM-3 and its ligands. TIM-3 exerts diverse immunoregulatory effects through its engagement with different ligands, which can (1) directly transmit inhibitory signals to attenuate immune activation, (2) synergize functionally with other coinhibitory receptors to refine cellular responses, or (3) facilitate broader immune modulation by interacting with alternative signaling pathways. Despite these insights, critical gaps remain in our understanding of the ligand-mediated regulation of TIM-3, including (1) the specific contextual factors that determine ligand-specific functions, (2) the possible existence of undiscovered TIM-3 ligands, and (3) the structural and mechanistic foundations of TIM-3-ligand interactions and downstream signaling events.

**Table:** **Table 1** Interaction and function of TIM-3 and its ligands in immune cells

Receptor/ligand	Cell type	Signaling pathway	Functional consequence
TIM-3/Gal-9	Th1	Galectin-9 induces intracellular calcium flux	Negatively regulate IFNγ secretion and suppress Th1 autoimmunity [18]
		Increased secretion of IL-10	Inhibiting Th1 effector function [64]
		Galectin-9 induced the decreased levels of proinflammatory cytokine	Regulate Th1 differentiation [65]
	Treg	Increased secretion of IL-10	Enhance the suppressive capacity of Treg cells [ 64, 66]
	CD8 ^+^ T-cell	PD-1 binds to Gal-9 and attenuates Gal-9/TIM-3-induced cell death	Induce CD8 ^+^ T-cell apoptosis [ 67, 68]
		Gal-9 expressed in MDSCs induces CD8 ^+^ T-cell exhaustion	Induce CD8 ^+^ T-cell exhaustion by MDSC [69]
	NK	MM-derived BMSCs can inhibit the immune response of NK cells	Promote NK cell exhaustion [70]
	Macrophages	AKT-GSK3β-IRF1pathway/STAT1-miR-155-SOCS1 signaling axis	Regulate M2 macrophage polarization [ 71– 73]
TIM-3/PtdSer	CD8 ^+^ T-cells	Tim-3 ^+^ APCs cooperates with PS on CD8 ^+^ T cells to limit antitumor immunity through T-cell trogocytosis and fratricide killing	TIM-3/PtdSer mediates T-cell trogocytosis [59]
	DCs	Tim-3 recognizes apoptotic cells through the FG loop in the IgV domain	Promote DCs’ function in phagocytosis of apoptotic cells and cross-presentation [58]
TIM-3/HMGB1	DCs	Toll-like receptors and cytosolic sensors	TIM-3/HMGB1 disrupts the recruitment of nucleic acids into DC endosomes to suppress DC maturation and function [13]
		cGAS-STING pathway	TIM-3/HMGB1 limits activation of the cGAS-STING pathway in intratumoral DCs [74]
TIM-3/CEACAM1	CD8 ^+^ T-cells	NF-κB/NLRP3/caspase-1 pathway	Induce CD8 ^+^ T-cell exhaustion [55]

### Regulatory network of TIM-3 ligands

The immunoregulatory function of TIM-3 relies on the binding of its ligands to the extracellular IgV domain, particularly the FG-CC′ cleft. Although Gal-9 and Ceacam1 bind to distinct regions within the IgV domain of TIM-3 [ 20, 36] , both the CEACAM1/TIM-3 interaction and the Gal-9/TIM-3 interaction trigger similar downstream events. These events involve the dissociation of Bat3, an inhibitory regulator of the TIM-3 signaling pathway, from its binding site on the TIM-3 cytoplasmic tail [ 20, 39] . Consequently, these two ligands may exert cooperative effects in regulating TIM-3 signaling. Notably, the HMGB1-binding epitope on TIM-3 significantly overlaps with the CEACAM1-binding epitopes at the FG-CC′ loop region within the IgV domain. The amino acid residue Q62 (E62 in humans) located in the FG-CC′ loop is pivotal in mediating interactions with both HMGB1 and CEACAM1. This observation prompts the intriguing question of whether competitive binding exists between HMGB1 and CEACAM1 for TIM-3. Currently, it remains uncertain whether this phenomenon indicates functional redundancy in TIM-3 signaling mediated by HMGB1 and CEACAM1 or if it represents a cell type-specific mode of ligand-receptor signaling. Among the ligands that interact with TIM-3, PtdSer is the only unique non-protein ligand. Mechanistically, PtdSer specifically binds to the FG-CC′ loops within the IgV domain of TIM-3. Given the overlapping binding sites located at the FG-CC′ loop within the TIM-3 IgV domain [ 24, 57] , elucidating the mechanisms by which CEACAM1, HMGB1, and other ligands coordinate their interactions with TIM-3 and subsequently modulate the functions of specific immune cell types is essential. A deeper understanding of this ligand network not only helps to reveal novel mechanisms of tumor immune evasion but also provides a theoretical foundation for the “multi-ligand synergistic blockade” strategy, which may become an important direction for future TIM-3-targeted therapies.

### Basic characteristics of TIM-3 and other novel checkpoints

Despite being the most effective cancer immunotherapy, both anti-PD-1/PD-L1 and anti-CTLA-4 regimens often encounter primary or acquired resistance in the majority of patients. Consequently, new immune checkpoint targets, such as TIM-3, Lymphocyte Activation Gene-3 (LAG-3), V-domain Ig Suppressor of T-Cell Activation (VISTA), T-cell Immunoreceptor with Ig and ITIM Domains (TIGIT), and Siglec-15, are being investigated either as combinatorial partners or alternative strategies to PD-1/PD-L1 blockade ( Table 2). These novel targets offer potential for broadening the therapeutic landscape of cancer immunotherapy [75]. Among these emerging immune checkpoint molecules, TIM-3 is a protein of considerable interest in both immunological and clinical research because of its complex role in health and disease. TIM-3 interacts with multiple ligands, each targeting different regions of its IgV domain. The TIM-3/Gal-9 interaction induces cell death in TIM-3 ^+^ Th1 cells [18]. Furthermore, CEACAM1 enhances the stability and inhibitory function of TIM-3 [ 20, 39] . TIM-3 also regulates innate immune responses through binding to PtdSer and HMGB1. When bound to PtdSer, TIM-3 promotes the recruitment of phagocytes for apoptotic cell clearance. In contrast, the interaction of HMGB1 with TIM-3 inhibits immune activation by blocking receptor interactions that promote pro-inflammatory signaling [ 13, 57] . Preclinical studies indicated that TIM-3 inhibitors exhibit efficacy comparable to PD-1 inhibitors in solid tumor models. Importantly, TILs that co-express TIM-3 and PD-1 exhibit a synergistic dysfunctional phenotype. Blocking TIM-3 and PD-1 may enhance anti-tumor immunity by restoring T-cell functionality [ 21, 64] . LAG-3 is a well-characterized type I transmembrane glycoprotein [64]. Multiple studies have shown that LAG-3 and PD-1 interact synergistically, contributing to T-cell exhaustion and inhibition through distinct signaling pathways. Dual blockade of PD-1 and LAG-3 has been shown to synergistically augment the effector functions of both CD8 ^+^ and CD4 ^+^ TILs [ 65– 67] . TIGIT, a co-inhibitory receptor, belongs to the expanding family of poliovirus receptor (PVR)-like proteins [68]. TIGIT plays a crucial role in maintaining immune tolerance and preventing excessive immune responses. It modulates the immune response to balance the immune system but also contributes to immune evasion in the tumor microenvironment [69]. In murine models, blockade of either PD-1 or TIGIT alone does not significantly inhibit the growth of CT26 tumors. However, dual blockade of PD-1 and TIGIT synergistically enhances the anti-tumor activity of CD8 ^+^ T cells, resulting in complete tumor rejection and prolonged survival in CT26 models. This effect is attributed to their complementary roles in immune regulation [ 70– 72] . VISTA, which stands for the V domain immunoglobulin suppressor of T-cell activation, is also pivotal in maintaining immune homeostasis. Recent studies have shown that blockade of VISTA via monoclonal antibodies enhances tumor-specific T-cell proliferation, infiltration, and cytokine production; reduces the accumulation of myeloid-derived suppressor cells; and suppresses Treg-mediated immunosuppression, thereby inhibiting tumor growth [73]. Furthermore, Siglec-15, a newly identified immune checkpoint in the SIGLEC superfamily, exhibits immunosuppressive characteristics in the tumor microenvironment and is currently under active investigation as a potential therapeutic target to increase the efficacy of cancer immunotherapy [74]. Siglec-15 on tumor-associated macrophages (TAMs) can actively inhibit the proliferation of antigen-specific T cells, thereby promoting a microenvironment that supports tumor growth [76]. Among these immune checkpoints, TIM-3 is recognized for its extensive role as a multifaceted immune regulator, modulating both innate and adaptive immune responses through cell type-specific interactions and context-dependent signaling pathways. Furthermore, comprehensive studies have elucidated the impact of TIM-3 on tumor progression and immune evasion, thereby providing a robust rationale for its clinical development. These characteristics collectively distinguish TIM-3 as a promising candidate among emerging immune checkpoints.

**Table:** **Table 2** Ligands for TIM-3, LAG-3, TIGIT, VISTIA, and SIGLEC-15 and their interactions

Immune checkpoint	Alternative name	Ligand	Expression	Mechanism of action	Major intervention
Tim-3	CD366, HAVCR2	Gal-9	APC, MDSC, CD4 ^+^ T cells	Gal-9 exerts its apoptotic effect on Th1 cells chiefly by stimulating calcium entry into their intracellular region	Sabatolimab (MBG453), Cobolima(TSR-022), BMS-986258, SYM023, TQB2618, INCAGN02390
		PtdSer	Apoptotic cells	TIM-3/PS mediates phagocyte recruitment for apoptotic cell clearance and potentiates antigen cross-presentation in TIM-3 ^+^ DCs
		HMGB-1	Cancer cells	Tim-3/HMGB-1 suppresses innate immune responses by recognizing nucleic acids through TLRs and cytosolic sensors
		CEACAM1	DCs, monocytes, macrophages, activated T cells	Regulate autoimmunity and anti-tumor immunity
LAG-3	CD223	MHCII	B cells, Mø, DCs, activated T cells	Inhibiting T-cell activation, proliferation, cytotoxicity, and cytokine production	Relatlimab, Fianlimab, Favezelimab, Ieramilimab, Encelimab, Miptenalimab, Tupastobart
		Galectin-3	Tumor cells, activated T cells, epithelial cells, macrophages, fibroblasts	Inhibiting anti-tumor T-cell response
		α-Synuclein	Neurons, muscle, heart, other tissues	LAG-3 recognizes α-synuclein fibrils, thereby modulating their endocytosis and intercellular transmission
		FGL1	FGL-1 protein secretion occurs primarily in hepatocytes	Inhibiting T-cell-mediated anti-tumor immunity
TIGIT	Vstm3, VSIG9, WUCAM	CD155	DCs, T cells, B cells, macrophages	Inhibiting T-cell activation and NK cell cytotoxicity	IBI939, Tiragolumab, COM902, EOS-448, HLX53 ASP8374, SAE-TGT, Domvanalimab
		CD112	Hematopoietic and nonhematopoietic tissues	Inhibiting the activation of T cells and NK cells
		CD113	Liver, lungs, testes, kidneys, and placenta	Inhibition of T-cell and NK cell activity
		Nectin-4	Tumor cells	Inhibiting NK cell activity
VISTA	PD-1H, VSIR, C10orf54, Gi24, B7-H5, SISP1, DD1α	PSGL1	Treg, NK, CD4 ^+^ T cells, CD8 ^+^ T cells, APC	Inhibiting T-cell function and proliferation	JNJ-61610588, CA170, W01080, KVA12123
		VISIG3	Tumor cells	VISTA-VSIG3 interaction inhibited T-cell proliferation and cytokine production
		Galectin-9	Tumor cells	Galectin-9 binding induces the apoptotic pathway through VISTA
Siglec-15	CD33	Sialyl-Tn	Tumor-associated macrophages, myeloid cells, osteoclasts, tumor cells	Siglec-15/Sialyl-Tn suppress the proliferation of antigen-specific T cells, thereby fostering a microenvironment conducive to tumor growth	NC318, AB-25E9, DS-1501

### Regulation of TIM-3 expression

TIM-3 is either constitutively or inducibly expressed on Th1 cells, CTLs, monocytes, macrophages, NKs, and DCs [ 41, 77] . The expression of TIM-3 on these cells is influenced by a variety of inducers. In this review, we summarize the potential regulatory mechanisms that affect TIM-3 expression.

#### Transcription factor

T-bet, known for its role in Th1 cell differentiation, has been identified as a regulator of TIM-3 expression during Th1 polarization and in DCs [78]. Yi *et al*. [79] suggested that the HCV core and gC1qR interaction induces T-bet, which subsequently enhances TIM-3 expression through the JNK pathway, thereby impairing monocyte/macrophage (M/Mφ) function during HCV infection. Additionally, TIM-3 expression is regulated by NFIL3, with the IL-27/NFIL3 signaling axis influencing effector T-cell responses by inducing TIM-3, IL-10, and T-cell dysfunction [80]. Signal Transducer and Activator of Transcription 3 (STAT3) is another pivotal transcription factor. Mishra *et al*. [81] utilized adoptive transfer experiments to elucidate the role of STAT3 in TIM-3 expression. They demonstrated that STAT3-driven upregulation of TIM-3 on DCs is crucial for the increased abundance of conventional DC subset 2 (cDC2) in chronically infected mice. Furthermore, the nuclear factor of activated T cells (NFAT) signaling pathway has been implicated in the regulation of TIM-3 expression in CD8 ^+^ T cells. Research indicates that NFAT-deficient CD8 ^+^ T cells fail to express effector cytokines or the inhibitory cell surface receptors PD-1, LAG3, and TIM-3 [82].

#### Cytokine

Wiener *et al*. [14] demonstrated that TGF-β1 induces TIM-3 promoter activity in HMC-1 cells. Yang *et al*. [83] reported that IL-12 upregulates TIM-3 expression on T cells independent of IFN-γ production and induces T-cell exhaustion. Boivin *et al*. [84] reported that IFN-β upregulates TIM-3 on the surface of Th1 cells in the absence of APCs. Hakim *et al*. [85] reported that IL-15 increases the expression of PD-1 and TIM-3 on CD4 ^+^ and CD8 ^+^ T cells and induces T-cell exhaustion. Zheng *et al*. [86] demonstrated that TNF-α can induce TIM-3 expression on NK cells via the NF-κB pathway, and elevated TIM-3 levels on NK cells indicate their dysfunction in human esophageal cancer. Zhao *et al*. [87] noted that IL-27 regulates the expression of TIM-3 and Blimp-1 through the STAT1 signaling pathway in Tregs. Importantly, our understanding of the mechanisms regulating TIM-3 expression on immune cells is incomplete and requires further in-depth investigation.

#### Post-translational regulation of TIM-3

TIM-3, a membrane-bound protein, is initially synthesized in the endoplasmic reticulum (ER) and subsequently transported to the cell surface, where it performs its inhibitory functions. This intricate journey involves sequential passage through the Golgi apparatus and secretory vesicles, facilitated by the protein-sorting machinery [88]. Upon arrival at the cell membrane, TIM-3 undergoes internalization and recycling, providing a rapid regulatory mechanism to modulate its surface abundance [89]. In recent years, the post-translational regulatory mechanisms controlling the expression of immune checkpoint molecules on the cell membrane have been increasingly elucidated, providing new opportunities for targeted therapeutic strategies with great clinical translational potential [4]. However, research on the post-translational modifications (PTMs) of TIM-3 remains scarce. We discuss several currently elucidated posttranslational regulatory mechanisms of TIM-3.

##### Glycosylation modification

The IgV domain and mucin domain of TIM-3 contain N-linked and O-linked glycosylation sites, respectively [36]. Gal-9 binds to N-linked glycans in the IgV domain of TIM-3, and this TIM-3/Gal-9 interaction depends on the glycosylation status of the IgV domain [90]. In addition, the glycosylation of TIM-3 is crucial for its interaction with CEACAM1, with their interaction mediating the suppression of effector T-cell function [20]. In contrast, Lee *et al*. [91] reported that non-glycosylated TIM-3-Ig fusion proteins expressed in bacteria bind to CD4 ^+^CD25 ^+^ T cells similarly to the glycosylated TIM-3-Ig protein produced in CHO cells. Recently, Vergoten *et al*. [92] employed molecular models to explore the role of the N-glycan in the interaction of TIM-3 with small-molecule ligands. They reported that N-glycosylation reinforces the interaction of TIM-3 with a small-molecule ligand, supporting the critical role of glycosylation in the binding of TIM-3 to its ligand.

##### Palmitoylation modification

Palmitoylation is a reversible PTM characterized by the attachment of a 16-carbon palmitoyl group to a protein cysteine residue via a thioester bond. This modification has been implicated in the regulation of the subcellular localization, stability, and function of modified proteins [93]. Our laboratory recently demonstrated that DHHC9 palmitoylates TIM-3 at cysteine 296, resulting in reduced degradation of TIM-3. Inhibition of DHHC9-mediated TIM-3 palmitoylation efficiently enhances the anti-tumor efficacy of chimeric antigen receptor T (CAR-T) cells and NK cells [94]. Furthermore, Cui *et al*. [95] reported that TIM-3 is also palmitoylated at cysteine 9. This palmitoylation of TIM-3 subsequently facilitates its binding to sortilin, directing TIM-3 to the lysosome for degradation. This process leads to diminished TIM-3 expression and dysfunction in decidual CD4 ^+^ T cells, ultimately leading to fetal loss [95]. The exploration of additional post-translational modifications of TIM-3 remains a critical avenue for future research.

### The function of TIM-3 in immune and tumor cells

#### T cells

TIM-3 was initially identified as a surface marker of Th1 cells, with Gal9 binding to TIM-3 to induce apoptosis in Th1 cells. Furthermore, TIM-3 plays an important role in regulating T-cell tolerance and suppressing Th1 and Th17 responses [ 18, 41] . During acute viral infection, TIM-3 expression is rapidly upregulated, facilitating the early differentiation of T-bet ^+^ effector T cells, which encompass both the CD4 ^+^ and CD8 ^+^ subsets [96]. In the context of cancer, TIM-3 specifically marks the most dysfunctional subset of tumor-infiltrating PD-1 ^+^ CD8 ^+^ T cells. These PD-1 ^+^TIM-3 ^+^ CD8 ^+^ T cells exhibited a reduced capacity to produce IFN-γ, TNF-α, and IL-2, indicating a phenotype of CD8 ^+^ T-cell exhaustion [ 49, 97– 99] . Blockade of TIM-3 and/or PD-1 using antibodies has been shown to enhance anti-cancer immunity and increase IFN-γ production in T cells [ 21, 100] . Conversely, overexpression of TIM-3 on T cells has been shown to accelerate tumor progression in an EL4 lymphoma mouse model [19]. In addition to its expression on Th1 and CD8 ^+^ T cells, TIM-3 is significantly upregulated on CD4 ^+^FoxP3 ^+^ Tregs in patients with chronic infections and cancer [101]. TIM-3 ^+^ Tregs represent potential therapeutic targets for anti-TIM-3 therapy in tumors, given their strong correlation with tumor severity and progression [102]. Liu *et al*. [103] demonstrated that TIM-3 blockade downregulates effector molecules of Tregs and inhibits tumor growth in a mouse model of head and neck cancer.

#### NK cells

TIM-3 is expressed on mature CD56 ^dim^CD16 ^+^ NK cells, whereas immature NK cells begin to express TIM-3 during maturation [6]. Li *et al*. [104] demonstrated that TIM-3 is expressed on more than 60% of decidual NK cells (dNKs). The Gal-9/TIM-3 signaling axis modulates the cytokine production and cytotoxicity of dNK cells at the maternal-fetal interface during early pregnancy. High TIM-3 expression indicates that NK cells have enhanced effector function, including increased cytotoxic capability and IFN-γ production. However, chronic stimulation induces excessive TIM-3 upregulation, leading to the emergence of exhausted or dysfunctional NK cells [ 51, 105] . We have reported that NK cell dysfunction is associated with high TIM-3 expression in both viral infections and tumor microenvironments, contributing to disease progression. Our previous research revealed a significant increase in TIM-3 expression on NK cells during CHB infection, where TIM-3 blockade has been shown to increase NK cell cytotoxicity [25]. Pires da Silva *et al*.[ 103, 106] reported that TIM-3 may serve as a marker of NK-cell exhaustion in advanced melanoma and that TIM-3 blockade reverses the exhausted phenotype of NK cells in melanoma. Our laboratory also previously reported that TIM-3 was significantly upregulated in both tumor-infiltrating liver-resident NK (LrNK) and conventional NK (cNK) cells and suppressed their cytokine secretion and cytotoxic activity. Mechanistically, the TIM-3/PtdSer axis inhibits Akt/mTORC1 signaling, thereby inducing NK cell dysfunction in HCC. TIM-3 blockade has been shown to inhibit HCC growth in an NK-cell-dependent manner [59]. In addition, elevated TIM-3 expression on NK cells may serve as a prognostic biomarker for disease progression. Our previous findings demonstrated that TIM-3 expression on NK cells plays a critical role in NK cell loss in atherosclerosis [107]. Taken together, these findings indicate that blocking TIM-3 on NK cells is a crucial strategy in tumor immunotherapy.

#### DCs

Recent studies have shown that TIM-3 is crucial in regulating DCs. In particular, type 1 conventional DCs (cDC1s), which are the most efficient at cross-presenting antigens and licensing naive CD8 ^+^ T cells, presented the highest levels of TIM-3 expression [ 108, 109] . Nakayama *et al*. [57] demonstrated that PtdSer binding to TIM-3 efficiently promoted the phagocytosis of apoptotic cells, enhancing antigen cross-presentation by TIM-3 ^+^ DCs to CD8 ^+^ T cells, which ultimately induced peripheral immune tolerance. The interaction between TIM-3 and HMGB1 disrupts the recruitment of nucleic acids into DC endosomes to suppress DC maturation and function. This suppression attenuates the therapeutic efficacy of DNA-based cancer vaccines and platinum-based chemotherapy [13]. TIM-3 also facilitates the differentiation of migratory DCs (migDCs) into mature regulatory DCs (mregDCs) by increasing reactive oxygen species (ROS) levels and activating the NLRP3 inflammasome [29]. The functional outcomes of TIM-3 signaling in DCs depend on both the specific subset of DCs and the availability of their cognate ligands. Studies have shown that targeting TIM-3 on DCs can enhance DC function and the response to paclitaxel chemotherapy in patients with breast cancer. Mechanistic investigations have indicated that blocking TIM-3 signaling in DCs increases the expression of Cxcl9 within intratumoral DCs [28]. Furthermore, TIM-3 blockade affects TIM-3 ^+^ DCs in a PtdSer-dependent manner, disrupting the trogocytosis of CD8 ^+^ T cells in melanoma [58]. Overall, TIM-3 serves as an inhibitory receptor on DCs, suggesting that modulating TIM-3 signaling in DCs has therapeutic potential for various clinical diseases.

#### Macrophage

TIM-3 is also expressed on macrophages and plays a major role in regulating their immunosuppressive effects. We previously discovered that TIM-3 expression significantly increases in both monocytes and tumor-associated macrophages (TAMs). Increased TIM-3 expression induces the activation and protumoral effects of TAMs in HCC, thereby promoting HCC development [26]. Additionally, our previous research demonstrated that TIM-3 promotes macrophage activation via the NF-κB/TNF-α pathway, thereby aggravating podocyte injury in diabetic nephropathy [110]. Previous studies demonstrated that TIM-3 blockade in macrophages upregulates TLR2/4 expression and enhances the production of pro-inflammatory cytokines, such as TNF-α and IL-6. This finding suggests that TIM-3 serves as a negative regulator of the TLR-mediated signaling pathway [ 111, 112] . Furthermore, TIM-3 promotes the polarization of M1/2-like macrophages. Specifically, TIM-3 signaling regulates M1 macrophage polarization by inhibiting the phosphorylation of IRF3, a downstream transcription factor of TLR4 [113]. Jiang *et al*. [114] reported that TIM-3 promotes M2 macrophage polarization by directly binding to the transcription factor signal transducer and activator of transcription 1 (STAT1) via residues Tyr256 and Tyr263 in its intracellular tail, thereby inhibiting the STAT1-miR-155-SOCS1 signaling axis. Ni *et al*. [115] demonstrated that the suppression of TIM-3/Gal-9 signaling effectively restrains M2 macrophage polarization in glioblastoma models. In addition to its immunosuppressive role, Wang *et al*. [116] demonstrated that TIM-3 inhibits the phagocytosis of *L*. *monocytogenes* by macrophages through its interaction with the transcription factor nuclear factor erythroid 2-related factor 2 (NRF2). Our studies have previously shown that TIM-3 negatively regulates the production of reactive oxygen species (ROS) and the secretion of pro-inflammatory cytokines IL-1β and IL-18 in macrophages. *TIM-3* knockout (KO) significantly exacerbates MCD-induced liver steatosis, whereas increased TIM-3 expression alleviates liver injury by modulating macrophage activation in MCD-induced non-alcoholic steatohepatitis (NASH) [117]. The regulatory role of TIM-3 in macrophage-mediated immune responses depends on disease type, and further research is needed to elucidate the complex roles of TIM-3.

#### Other immune cells

TIM-3 is constitutively expressed on mouse peritoneal mast cells, which are integral to host defense. In mast cells, TIM-3 enhances IgE ^+^ antigen-dependent cytokine secretion and cell survival. Anti-TIM-3 antibodies enhance the secretion of IL-3, IL-4, IL-6, and IL-13 in mast cells following IgE sensitization and antigen-dependent activation *in vitro*. These observations indicate that TIM-3 may contribute to the pathogenesis of autoimmune and allergic disorders by regulating mast cell activity [118]. Previous studies demonstrated that TIM-3 acts at a receptor-proximal site to enhance FcεRI-proximal signaling, thereby influencing mast cell activation [ 119, 120] . Additionally, Kimura *et al*. [31] used mouse models to analyze TIM-3 expression in microglia and identified TGFβ signaling as a key inducer. Conditional deletion of TIM-3 reprograms microglia into a neurodegenerative phenotype (MGnD), which reduces β-amyloid plaque deposition and improves cognitive function in 5× FAD mice. Furthermore, female APOE4 carriers with AD exhibit elevated TIM-3 expression and disrupted MGnD signaling. These findings highlight the critical role of TIM-3 in microglial function, particularly in APOE4-associated AD, and support the potential of TIM-3 inhibition as an effective therapeutic strategy for amyloid clearance and the mitigation of neurodegeneration. Further investigation is still necessary to decipher the role of TIM-3 signaling in other immune cells.

#### Tumor cells

In addition to its expression in immune cells, TIM-3 is also expressed in tumor cells. However, the mechanisms underlying the tumor-intrinsic role of TIM-3 in cancer progression and its implications for TIM-3-targeted therapies remain unclear. Shang *et al*. [121] reported that TIM-3 induces tumor cells to acquire characteristics of aggressive epithelial-mesenchymal transition (EMT) and may be involved in the pathogenesis of this malignancy. We previously demonstrated that TIM-3 is highly expressed in liver cancer cells. Overexpression of TIM-3 specifically in hepatocytes promotes tumor growth, whereas Tim-3 inhibition using antibodies or RNA interference (RNAi) suppresses tumor growth both *in vitro* and *in vivo*. Mechanistically, TIM-3 in hepatocytes activates NF-κB phosphorylation, which subsequently stimulates IL-6 secretion and STAT3 phosphorylation [122]. Notably, in breast cancer cells, TIM-3 promotes tumor aggressiveness and resistance to paclitaxel through the NF-κB/STAT3 signaling pathway [123]. TIM-3 is also highly expressed in drug-resistant glioma cells, and downregulation of TIM-3 expression remarkably reduces glioma cell resistance to temozolomide (TMZ) [124]. Guo *et al*. [125] reported that the glioma cell-intrinsic functions of the TIM-3/IL-6 signaling axis mediate the crosstalk feedback loop between glioma cells and tumor-associated macrophages (TAMs). Blocking this loop may provide a new therapeutic approach for glioblastoma multiforme (GBM). Schatton *et al*. [126] demonstrated that melanoma-specific TIM-3 overexpression attenuated tumorigenesis. Furthermore, TIM-3 blockade with antibodies inhibited the growth of immunogenic murine melanomas in hosts with intact T-cell populations. Collectively, these findings indicate that tumor cell-intrinsic TIM-3 promotes tumor invasion and subsequent metastasis, making it a crucial therapeutic target for cancer treatment.

### Expression of TIM-3 in different tumors and its clinical epidemiological data

We have summarized the expression of TIM-3 in different cancer types and its correlation with clinical outcomes, aiming to elucidate its potential as an independent prognostic biomarker. Thunyamon *et al*. [127] reported a significant association between elevated TIM-3 expression in acute myeloid leukemia (AML) patients and failure of initial 7 + 3 induction therapy, with complete remission (CR) rates of 16.2% compared with 36.4% in non-CR patients ( *P*  = 0.038). Hang *et al*. [128] demonstrated that TIM-3 positivity is significantly correlated with poorer recurrence-free survival (RFS) (hazard ratio [HR], 2.32; 95% confidence interval [CI], 1.44–3.73, *P* = 0.001) and overall survival (OS) (HR, 2.04; 95% CI, 1.29–3.20, *P* = 0.002). Furthermore, Wu *et al*. [129] reported that high TIM-3 mRNA expression was significantly associated with reduced PFS and OS in epithelial ovarian cancer patients (HR = 1.57, 95% CI = 1.29–1.91, *P*  < 0.001 and HR = 1.31, 95% CI = 1.06–1.63, *P* = 0.013, respectively). Chi *et al*. [130] assessed 248 patients with non-small cell lung cancer (NSCLC) and found that the OS of patients in the high TIM-3 expression group was shorter than that of patients in the low TIM-3 expression group ( *P* = 0.01). Patients with high TIM-3 and CD68/CD163 expressions had the worst prognosis, whereas patients with low expressions of both TIM-3 and CD68/CD163 presented the most favorable prognosis ( *P*  < 0.05).

### Targeting TIM-3 in tumor immunotherapy

Substantial evidence supports the comparable efficacy of targeting TIM-3 and PD-1 in preclinical research [131]. Ausejo-Mauleon *et al*. [132] demonstrated that TIM-3 is highly expressed in tumor cells, microglia, and macrophages in diffuse intrinsic pontine glioma (DIPG). Targeting TIM-3 in syngeneic models of DIPG prolongs survival and results in long-term survivors who are disease-free and exhibit immune memory. Koyama *et al*. [133] reported that treatment with PD-1 antibodies may lead to an increase in TIM-3 expression in the *in vivo* models of lung cancer, which is associated with adaptive resistance to PD-1 blockade. Immune checkpoint inhibitors have been reported to restore T-cell activity against cancer cells in HCC [ 134– 136] . Our previous studies demonstrated that targeting TIM-3 is an effective therapeutic strategy for HCC [ 26, 59, 122, 137] . Therefore, targeting both TIM-3 and PD-1 together is more effective than targeting either pathway alone. Notably, in preclinical cancer models, simultaneous blockade of PD-1 and TIM-3 has demonstrated a synergistic effect [138]. Sakuishi *et al*. [21] reported that concurrent targeting of the TIM-3 and PD-1 pathways can reverse T-cell exhaustion and restore anti-tumor immunity. Similarly, Fourcade *et al*. [49] reported that combined blockade of TIM-3 and PD-1 could ameliorate T-cell dysfunction in patients with melanoma.

TIM-3 functions as a critical immune checkpoint, and researchers have developed TIM-3-blocking antibodies. Both murine and human TIM-3 possess a common FG-CC′ cleft in the IgV domain, which serves as the binding site for PtdSer, CEACAM1, and HMGB1 [ 13, 20, 38] . The human IgG1 monoclonal antibody (mAb) LY3321367 effectively inhibits the binding of TIM-3 to PtdSer and partially disrupts its interaction with Gal-9. Hollebecque *et al*. [139] demonstrated that LY3300054 (an anti-PD-L1 mAb) in combination with LY3321367 exhibited superior clinical efficacy in the treatment of MSI-H/dMMR tumors in a phase I clinical trial.

In addition to anti-TIM-3 antibodies, TIM-3 small-molecule inhibitors have become a prominent area of focus, facilitating the development of a broad range of treatment strategies for immune checkpoint-based therapies. A small-molecule TIM-3 inhibitor was developed through fragment-based screening of TIM-3, employing nuclear magnetic resonance (NMR) spectroscopy for protein observation. However, no further functional validation has been conducted [140]. Another TIM-3 inhibitor, SMI402, effectively disrupts the binding of TIM-3 to CEACAM1, HMGB1 and PtdSer, and inhibits tumor growth *in vivo* [137]. However, the underlying mechanism remains unclear, and functional assays have been limited to the MC38 mouse tumor model.

Gal-9 primarily binds to the N-linked glycosylation region in the IgV domain, which is located on the opposite side of the FG-CC′ cleft [18]. Functional murine and human anti-TIM-3 antibodies target the binding sites of CEACAM1 and PtdSer but not Gal-9 [60]. Therefore, the PtdSer binding pocket of TIM-3 has emerged as an ideal target for the development of TIM-3 small-molecule inhibitors. Our laboratory previously identified a small molecule named ML-T7, which targets the FG-CC′ cleft of TIM-3 to disrupt its binding to PtdSer/CEACAM1. ML-T7 inhibits tumor progression by regulating the functions of T cells, NK cells, and DCs. Furthermore, ML-T7 can potentiate anti-PD-1-mediated anti-tumor responses [137]. Moreover, our laboratory also confirmed its therapeutic potential in preclinical models, warranting further translational research [141]. By targeting the PtdSer binding site of TIM-3, we developed a photoswitchable TIM-3 ligand termed photo-phosphatidylserine (phoPS), which mimics the function of PS. Upon exposure to light at wavelengths of 365 or 455 nanometers, the isomers of phoPS undergo periodic *cis*/ *trans* conformational transitions, thereby generating active and inactive forms of the TIM-3 ligand. This process facilitates the modulation of NK cell function both *in vitro* and *in vivo* [141]. We also utilized TIM-3 to develop a second-generation 4-1BB CD19 chimeric antigen receptor (CAR) linked with a T3/28 chimera, in which the extracellular domain of TIM-3 was fused with the CD28 transmembrane and cytoplasmic domains. The T3/28 chimera not only significantly prolonged the persistence of CAR-T cells *in vivo* but also conferred potent anti-tumor activity to T3/28 CAR-T cells in murine models, thereby providing novel insights into the optimization of adoptive T-cell therapies [142].

Currently, a substantial body of evidence suggests that targeting TIM-3, either with other checkpoint inhibitors or alone, has high potential for clinical benefit. In this review, we summarize several clinical trials using anti-TIM3 antibodies in cancer patients ( Table 3). TSR-022, a humanized anti-TIM-3 IgG4 antibody developed by Tesaro, was first identified by Tesaro [143]. TSR-022 has been evaluated both as a monotherapy and in combination with an anti-PD-1 antibody in human phase I/II clinical trials (NCT04139902, NCT06238635, NCT02817633, NCT03680508, and NCT03307785). Recently, Eli Lilly announced positive news regarding their antibody LY3321367, which specifically targets and inhibits TIM-3, having successfully completed phase I clinical trials. This achievement represents a significant milestone in their research and development pipeline. Furthermore, the anti-TIM-3 antibody MBG453 has been reported to have successfully completed a phase I clinical trial in the context of leukemia immunotherapy (TIM-3 in leukemia; immune response and beyond, 2021, NCT03066648). Additionally, several other anti-TIM-3 antibodies, including BGB-A425, BC3402, TQB2618, LB1410, and INCAGN02390, are currently undergoing phase 1/2 clinical trials (NCT05909904, NCT06111326, NCT06608940, NCT05834543, NCT05783921, NCT06010901, NCT05975645, NCT05645315, NCT05563480, NCT05400876, NCT05451407, NCT04370704, NCT05287113, NCT06056895, and NCT04463771). In the future, more basic research is needed to advance the development of anti-TIM-3 antibodies and enhance their application in clinical settings.

**Table:** **Table 3** Clinical trials of TIM-3 immune checkpoint inhibitors and their stages of development

Reagent name	Condition	Patientnumber	Phase	Manufacturer	Coblockade	Status	Identifier
TSR-022 TSR-042	Adult primary liver Cancer	42	II	University of Hawaii	Anti-PD-1	Recruiting	NCT03680508
TSR-022 TSR-042	Melanoma	62	II	Diwakar Davar	Anti-PD-1	Completed	NCT04139902
TSR-022 Dostarlimab	Cervical cancer	66	II	Meghan Shea	Anti-PD-1	Active, not recruiting	NCT06238635
TSR-022 TSR-042	Advanced or metastatic Cancer	58	I	Tesaro, Inc.	Anti-PD-1	Active, not recruiting	NCT03307785
MBG453 Azacitidine	Myelodysplastic Syndromes, Leukemia	530	III	Novartis Pharmaceuticals	Monotherapy	Active, not recruiting	NCT04266301
MBG453 PDR001 Decitabine	Advanced malignancies	252	I&II	Novartis Pharmaceuticals	Anti-PD-1	Terminated	NCT02608268
LY3321367 LY3300054	Solid tumor	209	I	Eli Lilly and Company	Anti-PD-L1	Completed	NCT03099109
MBG453 Pemigatinib INCAGN02385 INCAGN02390	Endometrial cancer	300	II	Incyte Corporation	FGFR, Anti-LAG3	Recruiting	NCT04463771
SYM023	Metastatic cancer, solid tumor, lymphoma	24	I	Symphogen A/S	Monotherapy	Completed	NCT03489343
Lomvastomig Tobemstomig Nivolumab	Advanced or metastatic esophageal squamous Cell carcinoma	210	II	Hoffmann-LaRoche	Anti-PD-1 Anti-LAG3	Active, not recruiting	NCT04785820
TSR-022 Nivolumab TSR-042 TSR-033 Docetaxel Pemetrexed Cisplatin Carboplatin	Neoplasms	475	I	Tesaro, Inc.	Anti-PD-1 Anti-LAG3	Recruiting	NCT02817633
BMS-986258 Nivolumab RHUPH20	Advanced cancer	92	I&II	Bristol-Myers Squibb	Anti-PD-1	Active, not recruiting	NCT03446040
Lomvastomig	Solid tumors, metastatic melanoma, non-small cell lung cancer, esophageal squamous cell carcinoma	134	I	Hoffmann-LaRoche	Anti-PD-1	Active, not recruiting	NCT03708328
MBG453 NIS793 Canakinumab	Myelodysplastic	33	I	Novartis Pharmaceuticals	Anti-TGF Anti-IL-1β	Recruiting	NCT04810611
MBG453 Decitabine PDR001 Azacitidine	Leukemia, myeloid, myelodysplastic syndromes, preleukemia	241	I	Novartis Pharmaceuticals	Anti-PD-1	Recruiting	NCT03066648
BGB-A425 Tislelizumab LBL-007	Head and neck squamous cell carcinoma	160	II	BeiGene	Anti-PD-1 Anti-LAG3	Active, not recruiting	NCT05909904
BC3402 Durvalumab	Hepatocellular carcinoma	83	I/II	Biocity Biopharmaceutics Co., Ltd	Anti-PD-L1	Active, not recruiting	NCT06111326
BC3402 Durvalumab Tremelimumab	Hepato cellular carcinoma (HCC)	43	I/II	Case Comprehensive Cancer Center	Anti-PD-L1 Anti-CTLA4	Not active	NCT06608940
TQB2618 Penpulimab Paclitaxel Cisplatin	Advanced esophageal squamous cell carcinoma	34	I/II	Chia Tai Tianqing Pharmaceutical Group Co., Ltd	Anti-PD-1	Active, not recruiting	NCT05834543
TQB2618 Penpulimab Paclitaxel Cisplatin or Carboplatin	Recurrent squamous cell carcinoma of the head and neck, metastatic squamous cell carcinoma	60	I/II	Chia Tai Tianqing Pharmaceutical Group Co., Ltd	Anti-PD-1	Active, not recruiting	NCT05783921
TQB2618 Penpulimab Anlotinib Hydrochloride capsules	Colorectal cancer	75	I	Chia Tai Tianqing Pharmaceutical Group Co., Ltd	Anti-PD-1	Active, not recruiting	NCT06010901
TQB2618 Penpulimab Anlotinib Hydrochloride capsules	Advanced hepatocellular carcinoma	29	I	Chia Tai Tianqing Pharmaceutical Group Co., Ltd	Anti-PD-1	Active, not recruiting	NCT05975645
TQB2618 TQB2450	Advanced solid tumor	127	I/II	Chia Tai Tianqing Pharmaceutical Group Co., Ltd	Anti-PD-L1	Recruiting	NCT05645315
TQB2618 Pempulimab Cisplatin Gemcitabine Hydrochloride injection	Nasopharyngeal carcinoma	90	II	Chia Tai Tianqing Pharmaceutical Group Co., Ltd	Anti-PD-1	Active, not recruiting	NCT05563480
TQB2618 Penpulimab	Relapsed/refractory lymphoma	92	I/II	Chia Tai Tianqing Pharmaceutical Group Co., Ltd	Anti-PD-1	Active, not recruiting	NCT05400876
TQB2618 Toripalimab	Melanoma	50	I	Chia Tai Tianqing Pharmaceutical Group Co., Ltd	Anti-PD-1	Active, not recruiting	NCT05451407
INCAGN02390 INCAGN02385 INCMGA00012	Melanoma	61	I/II	Incyte Corporation	Anti-LAG3 Anti-PD-1	Completed	NCT04370704
MBG453 INCAGN02390 INCAGN02385 Placebo	Head and neck cancer	176	II	Incyte Biosciences International Sàrl	Anti-LAG3	Completed	NCT05287113
MBG453 Verzistobart Tuparstobart	Unresectable clinical stage III Merkel cell carcinoma AJCC v8, Clinical stage IV Merkel cell carcinoma AJCC v8, Merkel cell carcinoma	12	II	University of Washington	Anti-LAG3	Active, not recruiting	NCT06056895

## Conclusion and Perspective

Immune checkpoint-targeted anti-tumor therapies have demonstrated promising clinical outcomes. Numerous clinical trials employing immune checkpoint inhibitors in cancer patients have reported satisfactory results. Nevertheless, only a small subset of patients achieve a complete response due to primary and adaptive resistance. Therefore, it is imperative to identify novel immune checkpoints and develop combination therapies to increase the proportion of patients who experience durable benefits. TIM-3, an immune checkpoint molecule, has emerged as a particularly promising target in the development of innovative anti-cancer therapeutic strategies. Investigating the inhibitory effects of TIM-3 and identifying an intervention approach are crucial for the development of targeted therapeutic strategies. The biology of TIM-3 is complex because of its broad expression across various immune cells, the presence of multiple ligands, and its unique signal transduction pathways [144].

TIM-3 is a negative regulator of immune responses and is expressed in a variety of cell types, including Th1/17 [ 18, 41] , effector T cells [97], Tregs [101], and innate immune cells [105]. We previously demonstrated that TIM-3 contributes to the progression of CHB and liver cancer by facilitating the functional exhaustion of CD8 ^+^ T cells and NK cells, as well as promoting the differentiation of macrophages into an M2-like phenotype [ 25, 26, 59, 117] . The current literature highlights the multifaceted roles of TIM-3 across diverse immune cell populations and its intricate regulatory mechanisms. This understanding provides new and profound insights into the strategic manipulation of TIM-3-targeted immune cells to augment anti-tumor immune responses. Notably, TIM-3 can function as either an inhibitory receptor or a stimulatory receptor, depending on the specific disease context. We previously reported that increased TIM-3 expression mitigates liver injury by modulating macrophage activation in MCD-induced NASH mice. Systemic inhibition of TIM-3 might disrupt immune tolerance mechanisms, thereby increasing the risk of autoimmune reactions, such as colitis and pneumonitis, or impairing tissue homeostasis. Prolonged TIM-3 blockade may further exacerbate inflammatory conditions by dysregulating immune cell function in peripheral tissues. These potential risks underscore the need for tissue-specific targeting strategies, meticulous patient selection based on TIM-3 expression profiles, and rigorous monitoring for autoimmune or off-target effects during clinical trials. Therefore, the comprehensive mechanisms and pathways of TIM-3 blockade are currently a focal point of research.

Increasing evidence suggests that targeting TIM-3 along with other checkpoint inhibitors holds significant promise for developing therapeutic modalities with durable clinical benefits. The IgV domain of murine and human TIM-3 contains a conserved FG-CC′ cleft, which serves as the binding site for PtdSer, CEACAM1, and HMGB1 [ 13, 20, 24, 36– 38] . The TIM-3 blocking antibody primarily targets the FG-CC′ cleft, thereby inhibiting the interaction between TIM-3 and its ligands, thus exerting its functional efficacy [60]. The ligands of TIM-3, including CEACAM1, HMGB1, and PtdSer, share overlapping binding sites at the FG-CC′ cleft. It is necessary to understand whether these ligands coordinate their interactions with TIM-3. Importantly, the PtdSer binding pocket of TIM-3 is a promising target for the development of TIM-3 inhibitors, which has significant implications for the development of new therapeutic strategies for relevant diseases. We employed a virtual screening strategy that targets the FG-CC′ cleft of TIM-3 to disrupt its interaction with PtdSer/CEACAM1. This approach led to the successful identification of ML-T7, a compound that enhances the function of CD8 ^+^ CTLs, NK cells, and DCs, thereby enhancing tumor immunotherapy and potentiating the anti-tumor responses induced by anti-PD-1 therapy [137]. Therefore, understanding the ligands of TIM-3 and their binding patterns would benefit the future development of TIM-3-related inhibitors. Further research is warranted to explore additional potential ligands. Additionally, TIM-3 interacts with multiple ligands, some of which also engage with other immune receptors. This redundancy may result in the activation of compensatory pathways when TIM-3 is inhibited, potentially diminishing therapeutic efficacy or contributing to resistance. For instance, blocking TIM-3 could cause ligands to bind to alternative receptors, thereby maintaining immunosuppressive signaling in the tumor microenvironment.

Currently, a variety of TIM-3-targeted immunotherapeutic agents are undergoing phase 1/2 clinical trials. These agents include cobolimab, sabatolimab, BGB-A425, BC3402, TQB2618, NB002, AZD7789, LB1410, and INCAGN02390. However, anti-TIM-3 monoclonal therapy has not demonstrated significant therapeutic effects. The enrolled patients ( *n* = 219) had a range of cancers, most commonly ovarian (17%) and colorectal cancer (7%); patients received sabatolimab ( *n* = 133) or sabatolimab plus spartalizumab ( *n* = 86). The MTD was not reached. The most common adverse event suspected to be treatment-related is fatigue (9%, sabatolimab; 15%, combination). No responses were observed with sabatolimab [145]. In the STIMULUS clinical trial, researchers administered sabatolimab in combination with a hypomethylating agent (HMA) to a cohort comprising 53 patients with high- or very high-risk myelodysplastic syndrome (HR/vHR-MDS) and 15 patients with chronic myelomonocytic leukemia (CMML). A study of HR/vHR-MDS patients identified thrombocytopenia and constipation as the two most prevalent adverse effects, each occurring in 56.6% of patients, with nausea following at an incidence of 54.7%. Additionally, seven patients experienced immune-related adverse events. Among the 51 evaluable HR/vHR-MDS patients, the median duration of response was 17.1 months, with an overall response rate of 56.9%. For CMML patients, the median duration of response is 5.6 months, and the overall response rate is 66.7% [146]. In the future, it will be necessary to develop more effective TIM-3 inhibitors and explore combination strategies involving TIM-3 and other immune checkpoints. It is imperative for researchers to elucidate the precise mechanisms by which TIM-3 interacts with its ligands, such as Gal-9, and how this interaction regulates immune cell function. For example, the application of X-ray crystallography and cryo-electron microscopy techniques facilitates the determination of the three-dimensional structure of TIM-3 in complex with its ligands, thereby providing valuable insights for drug design. High-throughput screening methods can be utilized to identify potential lead compounds for TIM-3 inhibitors. Prior to advancing to clinical trials, the efficacy of TIM-3 inhibitors should be validated in appropriate animal models. Furthermore, blocking both TIM-3 and PD-1 can synergistically enhance T-cell activation and proliferation, resulting in a more robust anti-tumor immune response. It is imperative for researchers to elucidate the molecular pathways through which these immune checkpoints interact and to understand how their combined blockade can effectively overcome immune suppression.

In summary, a comprehensive understanding of the functional characteristics of TIM-3 holds promise for the development of novel therapeutic strategies for a variety of diseases. However, current knowledge regarding the structure, function, and expression of TIM-3 across different cancer types remains limited and requires further investigation.
